# Top-down resolution of visual ambiguity – knowledge from the future or footprints from the past?

**DOI:** 10.1371/journal.pone.0258667

**Published:** 2021-10-21

**Authors:** Jürgen Kornmeier, Kriti Bhatia, Ellen Joos

**Affiliations:** 1 Institute for Frontier Areas of Psychology and Mental Health, Freiburg, Germany; 2 Department of Psychiatry and Psychotherapy, Medical Center, University of Freiburg, Freiburg, Germany; 3 Faculty of Medicine, Freiburg, Germany; 4 Experimental Cognitive Science, Eberhard Karls University Tübingen, Tübingen, Germany; 5 INSERM U1114, Cognitive Neuropsychology and Pathophysiology of Schizophrenia, University of Strasbourg, Strasbourg, France; Technical University of Madrid, SPAIN

## Abstract

Current theories about visual perception assume that our perceptual system weights the a priori incomplete, noisy and ambiguous sensory information with previous, memorized perceptual experiences in order to construct stable and reliable percepts. These theories are supported by numerous experimental findings. Theories about precognition have an opposite point of view. They assume that information from the future can have influence on perception, thoughts, and behavior. Several experimental studies provide evidence for precognition effects, other studies found no such effects. One problem may be that the vast majority of precognition paradigms did not systematically control for potential effects from the perceptual history. In the present study, we presented ambiguous Necker cube stimuli and disambiguated cube variants and systematically tested in two separate experiments whether perception of a currently observed ambiguous Necker cube stimulus can be influenced by a disambiguated cube variant, presented in the immediate perceptual past (perceptual history effects) and/or in the immediate perceptual future (precognition effects). We found perceptual history effects, which partly depended on the length of the perceptual history trace but were independent of the perceptual future. Results from some individual participants suggest on the first glance a precognition pattern, but results from our second experiment make a perceptual history explanation more probable. On the group level, no precognition effects were statistically indicated. The perceptual history effects found in the present study are in confirmation with related studies from the literature. The precognition analysis revealed some interesting individual patterns, which however did not allow for general conclusions. Overall, the present study demonstrates that any future experiment about sensory or extrasensory perception urgently needs to control for potential perceptual history effects and that temporal aspects of stimulus presentation are of high relevance.

## Introduction

Our everyday experience suggests that we perceive the world as it is. However, due to the limited capacities of our senses the available information is a priori remarkably incomplete, noisy, and to varying degrees ambiguous. The perceptual system has to construct– as fast as possible–stable and reliable perceptual interpretations out of this limited information. One strategy to overcome this perceptual inference problem, as already described by Helmholtz and others (for a nice historic overview see [1]), is to take temporal aspects of perception–on different time scales– into account. Our perceptual environment changes only slightly from one moment to the next. We can thus exploit perceived regularities from our immediate perceptual history to make predictions about the immediate perceptual future. But also longer-term memories can influence how we perceive the world. In the recent years, predictive coding theories have successfully modeled the influence of the perceptual history for normal visual perception and altered perception in psychiatric disorders [2–5].

Different lines of research demonstrate the influence of the perceptual history from different time scales on the current percept. Studies reported positive effects (i.e. current stimuli are likely to be perceived in the same way as previous ones), like positive priming [6–9], positive hysteresis [10,11] or serial dependence [12–14]. In contrast, other studies reported negative effects (e.g. current stimuli are likely to be perceived as opposite of the previous one), like adaptation or negative hysteresis [10,15–17]. Further examples are motion aftereffects [18], contrast aftereffects [15], or repetition suppression [19]. Moreover, perceptual history effects can be found at different time scales and different complexity levels along the perceptual processing chain, from low-level visual functions, like contrast or motion perception, up to the processing of emotional content of faces and even beyond [10,20–22]. Moreover, the perceptual history not only influences our current percepts, but our current percepts also add to our perceptual memories and change them. We thus never process the same sensory input in the same way [e.g. 23,24].

### Long-term perceptual history effects

**Fig 1:**
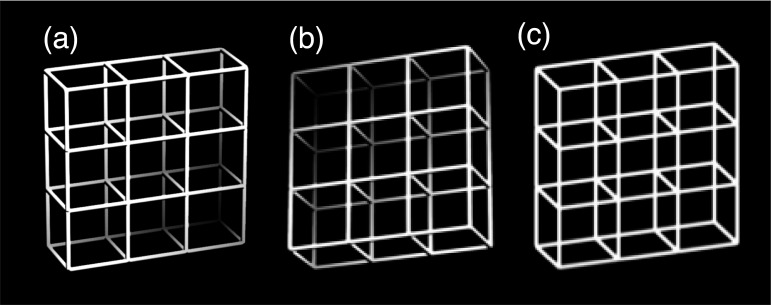
(a) Disambiguated lattice variant with the front-side to the bottom-right (B-perspective); (b) disambiguated variant with the front-side to the top left (T-perspective). (c) Necker lattice, composed of 3x3 Necker cubes, as introduced by Kornmeier et al. (2001).

**Fig 2:**
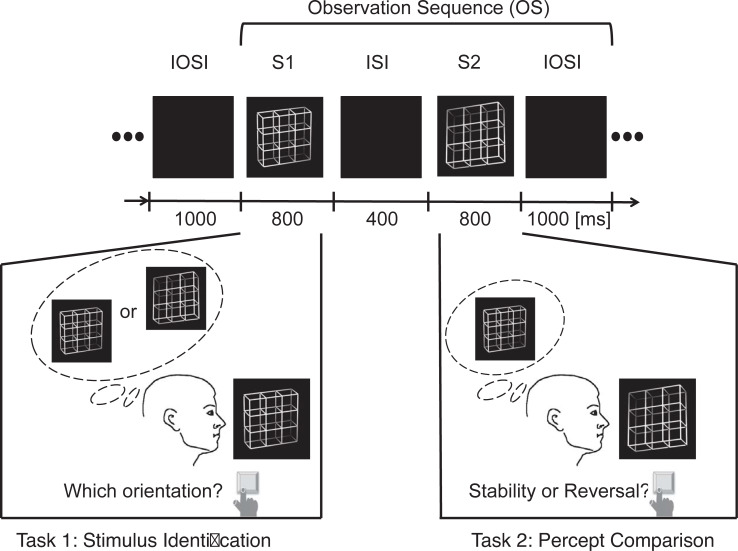
Experimental task. Lattice stimuli were presented in pairs, the first lattice stimulus S1 was followed by the second lattice stimulus S2. S1 was presented for 800 ms and participants indicated with different keys whether they perceived it in the B- or the T-perspective. After a grey screen inter-stimulus interval (ISI) of 400 ms lattice S2 was presented for 800 ms. Participants compared their percept of S2 with their memorized percept of S1 and indicated by key press either perceptual stability (same perceived perspectives of S1 and S2) or perceptual reversal (change between B- and T-perspective). S2 was followed by an inter-observation interval (IOSI) of 1000 ms. In the present example case, both S1 and S2 were disambiguated lattice versions. In our study, the ambiguity levels of S1 and S2 stayed constant within but varied between experimental conditions. A variant of this figure has already been presented in a recent publication from our lab [see Fig 2 in 61].

One convincing example of how our perceptual memories contribute to our current percept is 3D perception and in particular, perception of the ambiguous Necker cube [25]: During our first steps of visual processing, images of three-dimensional objects are projected on two-dimensional retinae [e.g. 26]. We thus only have direct access to two out of three dimensions. Our perceptual system needs to reconstruct the missing third dimension out of secondary information, like occlusion, stereopsis, movement parallax, etc. [e.g. 27]. The Necker cube (see the Necker lattice, i.e. a construction of 3x3 Necker cubes in Fig 1C) is a 2D drawing of a 3D cube grid that demonstrates the limits of such perceptual 3D reconstruction. The 2D image on our retinae induced by the Necker cube drawing is equally compatible with two 90° angle cubes with opposite spatial orientations. As a consequence, perception of the Necker cube is unstable and reverses between a “front-side to the bottom-right” perspective (“B”-perspective, Fig 1A) and a “front-side to the top left” perspective (“T”-perspective, Fig 1B). Moreover, most observers’ percepts are biased towards a B-perspective (Fig 1A) [9–11,28]. One post-hoc explanation of this bias is close to Helmholtz’s (and others) perceptual inference approach and concurrently demonstrates influences from long-term perceptual memory: During our lifetime we much more often look down on objects in our environment than up to them. This makes the B-perspective of the Necker cube more probable than a T-perspective interpretation. In the following, we will call this the long-term perceptual history effect. Even more confirmation for long-term memory effects comes from the fact that the Necker cube image on our retinae is in principle compatible with almost infinitively many geometric objects [for a nice demonstration of this see Fig 2B in 29] and the common view is that 90° interpretations are simply more probable because of the many 90° objects in our environment.

### Immediate perceptual history effects

The Necker cube example can also nicely demonstrate how the immediate perceptual history can influence our current percept. Having observed a disambiguated cube variant can have priming (identical percept) or adaptation effects (opposite percept) for the subsequent perception of the Necker cube, depending on how long the preceding disambiguated variant had been observed [e.g. 8]. Furthermore, it has been shown that with a discontinuous presentation mode (Necker cube presentations are interrupted by short blank screen gaps) the dynamics of perceptual reversals [i.e. its “dwell time”, e.g. 30] strongly depend on the duration of the intermediate gap [31–36].

### Precognition effects

Precognition is the proposed ability to perceive or sense events or more general information at the present moment, although this information will only be generated in the future. A large body of phenomenal reports about precognition experiences [37–39], together with a number of empirical studies and meta-analyses thereof, indicate the existence of precognition phenomena and have even evoked hypotheses about the relevance of precognition during the evolution of human cognition [40,41]. On the other hand, phenomenal reports cannot be confirmed statistically and replications of many of the empirical studies failed to reproduce the effect, taking the existence of precognition into question [41–50].

One critical factor for precognition experiments is randomization. The stimulus material needs to be presented in a randomized order to prevent any regular temporal pattern of stimulus presentation that may allow predicting the future stimuli and thus confound the study results [e.g. 51]. A second aspect of an appropriate randomization is making sure that all used stimuli are presented about equally often over the whole experiment in order to prevent possible quantitative confounds in the results. However, presenting different types of stimuli in a precognition experiment, using an appropriate randomization procedure and concurrently making sure that each stimulus occurs with about the same frequency, may not be enough control for precognition experiments. Another potentially confounding but so far hardly recognized factor in previous precognition studies is the influence of the perceptual history. As discussed above, it has been shown that what we see at the current moment is influenced by what we saw a millisecond, a second, a minute (or even longer) before. And even with a randomization procedure it is possible that certain stimuli have in some way stronger and/or longer lasting memory effects on the perceptual system than other stimuli. It is thus mandatory for any precognition analysis to control for perceptual history effects.

Ambiguous figures, like the Necker cube, are regarded as interesting stimuli to study precognition phenomena, because observing an ambiguous figure evokes unstable perceptual states [52,53]. The idea is that observers in unstable perceptual states or more generally unstable mental states are more sensitive for the proposed information from the future than observers in stable perceptual and/or mental states [41,54–56]. Some years ago, Bierman [54] studied precognition effects, using the ambiguous Necker cube and two apparently disambiguated cube variants, corresponding to the two most probable Necker cube percepts. His participants observed a continuous presentation of the Necker cube and were instructed to press one key when perceiving the B-perspective (Response 1) and another key when perception changed to the T-perspective (Response 2). Immediately after Response 2, the computer program replaced the Necker cube with a random selection of one of the two disambiguated cube variants. Bierman compared the duration of the B-perspective during observation of the ambiguous Necker cube (i.e. the time between Response 1 and Response 2) as a function of the identity of the subsequent disambiguated variant, presented after Response 2. The basic assumption underlying this study was that the two disambiguated cube variants retrocausally or precognitively influence the duration of the B-perspective perception of the preceding Necker cube in different directions. Bierman executed three separate experiments in this way, a pilot (N = 5 participants) and two follow-up experiments with N = 26 and N = 122. According to his description, the pilot experiment served as hypothesis generating, and its results indicated a retrocausal adaptation effect. In detail the duration of the B-perspective percept was shorter when a disambiguated T-perspective cube followed, compared to a subsequent disambiguated B-perspective cube variant. In Experiment 2, he found a weak confirmation of this result, in Experiment 3 only a tendency.

The study described above, contained a number of obscurities. It was unclear, why Bierman restricted his analyses only to the duration of B-perspective Necker cube percepts, how and at exactly what time (before or after execution of the experiment) the criteria for outliers had been defined (particularly in the pilot experiment) and why he integrated the data from the hypothesis-generating pilot experiment into the confirmatory final analysis, to name only some. Moreover, and particularly relevant for the present study, Bierman interpreted his results as evidence for perceptual precognition without controlling for perceptual history effects.

In the present study, we also presented Necker cube stimuli and disambiguated stimulus variants, but used a slightly different experimental paradigm and a different analysis approach than Bierman (2011), as will be motivated below. Overall, we executed two experiments, where we systematically investigated both potential influences from the perceptual future but also potential influences from the perceptual past. In summary, in our Experiment 1, we found strong evidence for perceptual history effects, some indication for precognition-like effects in individual participants but no significant precognition effects on the group level. In the subsequent Experiment 2, we again found perceptual history effects and some participants with precognition-like effect indications but no precognition effects on the group level.

## Methods– Experiment 1

### Participants

13 participants (7 women, 6 men) took part in Experiment 1. The median age was 24 with a range between 21 and 34 years. All participants had normal or corrected-to-normal visual acuity [57] and gave their written consent to participate in this study. The study was performed in accordance with the ethical standards laid down in the Declaration of Helsinki [58] and was approved by the local ethics board (Ethik-Kommission der Albert-Ludwigs-Universität Freiburg).

### Stimuli

We used a so-called Necker lattice [59,60], which is a variant of the famous Necker cube [25] consisting of 3x3 Necker cubes (Fig 1C). This Necker lattice is most often perceived in two different and mutual exclusive 3D configurations, either with the front side pointing to the bottom right (B-perspective) or with the front side pointing to the top left (T-perspective). We also used two disambiguated lattice variants, corresponding to these two perceptual interpretations of the Necker lattice (Fig 1A and 1B). These disambiguated lattices (Fig 1B and 1C) were transparent like the Necker lattices but contained depth cues, based on a drawing model incorporating shading, central projection, and aerial perspective (OpenGL lighting model; [61]).

All lattices had light grey edges on a dark background and were presented with a visual angle of 7.5° x 7.5°. The Necker lattice had a uniform luminance of 40 cd/m^2^. Due to the depth cues of the disambiguated lattice, the edges had inhomogeneous luminance with darker edges of the apparent back layer than of the apparent front layer. We chose the edge luminance of those lattices in a way that the average luminance across the eight outer lattice corners was at 40 cd/m^2^. The stimuli were presented with the open software PsychoPy [62] on an Apple Mac mini computer.

### Paradigm

Participants had a distance of 114 cm from a Philips GD 402 monochrome CRT screen (refresh rate = 85 Hz, screen resolution = 800 x 600 pixel) and were instructed to fixate a cross in the middle of the screen. Experiment 1 consisted of four conditions. Within each condition, the lattice stimuli were presented in pairs forming a so-called observation sequence (“OS”) in the following way (see also Fig 2): The first stimulus S1 was presented for 800 ms, followed by an inter-stimulus interval (“ISI”) with a grey screen for 400 ms, which was followed by stimulus S2 for 800 ms and finally by another grey screen (inter-observation-sequence interval, “IOSI”) for 1000 ms. Within each OS, participants had two tasks. In Task 1 they indicated their percept of the presented lattice stimulus S1 per key press (different keys for B-perspective and T-perspective). In Task 2 they compared their percept of stimulus S2 with their previous percept of S1 and indicated a changed percept by one key and perceptual stability (i.e. identical percepts of S1 and S2) by a second key. In the conditions with the ambiguous lattice stimuli, perceptual reversals occur endogenously within the perceptual system of the observer. According to the literature, such endogenous reversals take place with a probability of 0.3 on average (e.g. [31,63]). The disambiguated lattices were alternated pseudo-randomly to simulate the spontaneous perceptual reversals of the ambiguous variants. We adopted the reversal probability from ambiguous variants for the exogenous reversal rate of the disambiguated Necker lattice variants.

**Fig 3:**
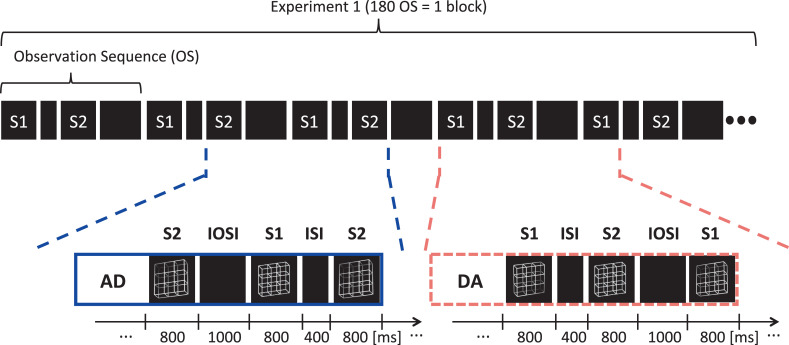
Experimental conditions–schematic overview. In the present analysis of Experiment 1 we restricted the analysis to two out of four experimental conditions (AD and DA) where ambiguous lattices alternated continuously with disambiguated lattice variants. The conditions were subdivided into three experimental blocks of about 9 min duration. Each experimental block was composed of 180 repetitions of observation sequences (OS). Within one OS two lattice stimuli S1 and S2 were presented in succession and participants had to execute respective tasks, as explained in Fig 2. In Condition AD, stimulus 1 (S1) was always an ambiguous lattice (“A”) and S2 a disambiguated lattice variant (“D”). In Condition DA, S1 was the disambiguated lattice and S2 the ambiguous lattice variant. Lattice ambiguity levels stayed constant within but differed between experimental blocks/conditions. A variant of this figure has already been presented in a recent publication from our lab [see Fig 3 in 61].

Each condition was subdivided in 3 experimental blocks of about 9 minutes duration and each experimental block consisted of 180 observation sequences from the respective condition (Fig 3). We varied the ambiguity levels of S1 and S2 between experimental conditions but kept them constant within experimental conditions (and thus within experimental blocks). This resulted in four experimental conditions as defined in Table 1.

**Table 1:** Experimental condition.

Conditions	Ambiguity level of S1 and S2
**Condition 1: AA**	S1 and S2 ambiguous
**Condition 2: AD**	S1 ambiguous; S2 disambiguated
**Condition 3: DA**	S1 disambiguated; S2 ambiguous
**Condition 4: DD**	S1 and S2 disambiguated

A = ambiguous; D = disambiguated.

In total, each participant thus performed 12 experimental blocks within three successive time periods with breaks of about 10 minutes between periods. The order of the experimental blocks was pseudo-randomized. One experimental session lasted for about 3 hours with roughly 1.5 hours measurement time.

The study described here was part of a larger research project and included EEG recordings. Separate and non-overlapping aspects of this study have already been published elsewhere [64]. In the present analysis, we only focused on the behavioral data from the conditions AD and DA, in which ambiguous Necker lattices alternate with disambiguated lattice stimuli. The here analyzed aspects of the study data are novel and had not been part of the above mentioned publication [64].

### Data analysis

#### Perceptual history effects

When focusing on Condition AD to study the influence of the immediate perceptual history on the current percept of an ambiguous Necker lattice, we sorted the observation sequences (OS) into eight separate groups of stimulus/percept sequences:

BBB–Sequences (AD Condition):

- The disambiguated lattice S2 from the previous OS had a **B**-perspective

- The currently observed ambiguous lattice S1 from the current OS is perceived in the **B**-perspective
- The disambiguated lattice S2 from the current OS will have a **B**-perspective

BBT–Sequences:

- The disambiguated lattice S2 from the previous OS had a **B**-perspective
- The currently observed ambiguous lattice S1 from the current OS is perceived in the **B**-perspective
- The disambiguated lattice S2 from the current OS will have a **T**-perspective

**Fig 4:**
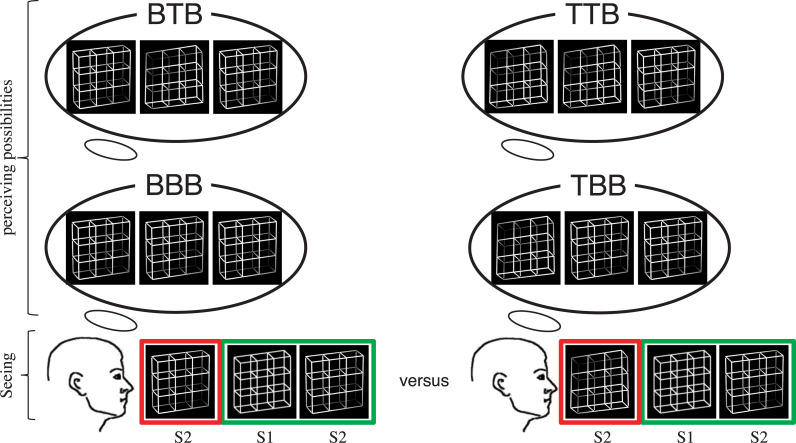
Grouping of analysis sequences. The logic behind the grouping underlying the data analysis, exemplified for the history effect analysis. We compared the percentage of group occurrences of percepts of the ambiguous stimulus (S1 current OS, green frame) given that the subsequently presented lattice variants will be identical but the preceding lattice variants differed. For the precognition effect analysis we varied the ambiguity level of the subsequent stimulus but kept it for the preceding stimuli constant.: P-perspective; T: T-perspective.

The logic of this grouping is visualized in Fig 4. According to this rule the other six groups were BTB, BTT, TBB, TBT, TTB and TTT. The number of occurrences of either of those groups will be labeled with an “n”-prefix (e.g. nBTB = number of BTB group occurrences). The average number of analyzed sequences across participants is reported in Table 2.

**Table 2:** Average numbers of sequences.

	Condition 2 (AD)	Condition 3 (DA)
**BBB**	77.54 (±5.62)	66.46 (±6.17)
**BBT**	73.23 (±5.34)	70.462 (±5.72)
**TBB**	81.39 (±5.66)	90.385 (±7.6)
**TBT**	81.23 (±5.86)	89.231 (±8.3)
**BTB**	57.54 (±4.88)	55.62 (±5.98)
**BTT**	62 (±5.34)	50.92 (±5.91)
**TTB**	42.15 (±7.26)	42.85 (±5.14)
**TTT**	41.85 (±7.67)	47.46 (±6.42)

Average number of sequences across participants (± SEM).

A similar logic applies when focusing on Condition 3 (DA), again with eight separate groups of sequences:

BBB–Sequences (DA Condition):

- The disambiguated lattice S1 from the current OS had a **B**-perspective
- The currently observed ambiguous lattice S2 from the current OS is perceived in the **B**-perspective
- The disambiguated lattice S1 from the subsequent OS will have a **B**-perspective

BBT–Sequences:

- The disambiguated lattice S1 from the current OS had a **B**-perspective
- The currently observed ambiguous lattice S2 from the current OS is perceived in the **B**-perspective
- The disambiguated lattice S1 from the subsequent OS will have a **T**-perspective

According to this rule the other six groups were BTB, BTT, TBB, TBT, TTB and TTT. The number of occurrences of those groups will be labeled with an “n”-prefix (e.g. nBTB = number of BTB group occurrences). The average number of analyzed sequences across participants is reported in Table 2.

**Fig 5:**
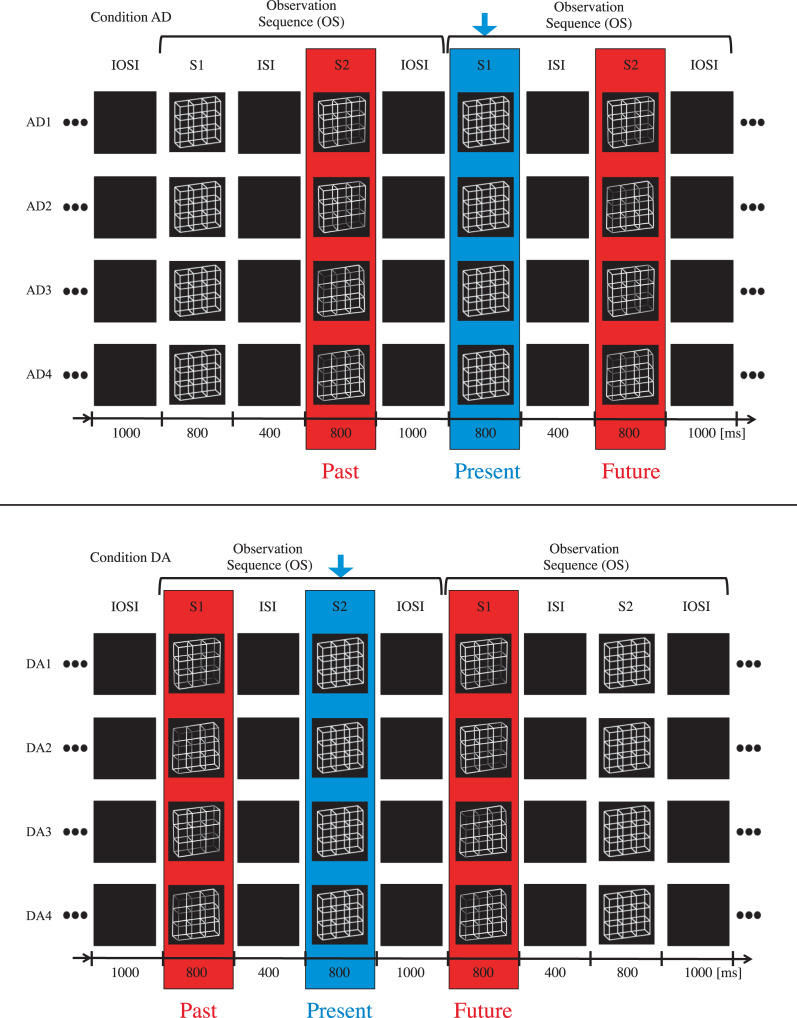
Groups of sequences. Top: From Condition AD we selected separate groups of observation sequences (OS) to analyze precognition effects while controlling for the perceptual history and vice versa. The history effects contrasts compared group (AD1) with group (AD3) and group (AD2) with group (AD4). The precognition effect contrasts compared group (AD1) with group (AD2) and group (AD3) with group (AD4). The blue arrow indicates what participants observed at a current moment. Bottom: Grouping of Condition DA according to the same logic.

Please keep in mind that in the AD condition the temporal distance between onset of the ambiguous lattice and the preceding disambiguated lattice variant was 1000 ms whereas in the DA condition it was only 400 ms and thus 600 ms shorter (see Fig 5). Accordingly, the temporal distance between onset of the ambiguous lattice and the subsequent disambiguated lattice variant in the AD condition was 400 ms whereas in the DA condition it was 1000 ms and thus 600 ms longer.

Further, the tasks differed between stimuli S1 and S2 (see Fig 2), which results in another difference between the AD and DA data in terms of the relation between stimulus ambiguity level and task. In condition AD, participants had to perform a comparison task in response to disambiguated variants (S2) and an identification task in response to ambiguous variants (S1). In condition DA, however, participants had to perform an identification task in response to disambiguated variants (S1) and a comparison task in response to ambiguous variants (S2).

In order to study perceptual history effects and precognition effects, we first calculated probabilities of B-percepts of the ambiguous Necker lattice as a function of the identity of the preceding and subsequent disambiguated lattice variants in the following way (the bold letters indicates the perceptual interpretation of the ambiguous Necker lattice):

$$P(BBB)=\frac{nBBB}{nBBB+nBTB}$$ (1)

$$P(BBT)=\frac{nBBT}{nBBT+nBTT}$$ (2)

$$P(TBB)=\frac{nTBB}{nTBB+nTTB}$$ (3)

$$P(TBT)=\frac{nTBT}{nTBT+nTTT}$$ (4)

We restricted our analysis to B-percepts of the ambiguous Necker lattice for the following reason: Focusing on Eq (1) it becomes immediately clear that P(BBB) + P(BTB) = 1. The same principle applies to Eqs 2–4. The probabilities of T-percepts of the Necker lattice as a function of preceding and subsequent disambiguated Necker lattice are thus completely dependent on the probabilities of the B-percepts.

For the statistical analyses of perceptual history and precognition effects, we calculated an ANOVA with the factors CONDITION, (immediate) HISTORY and (immediate) FUTURE, each with two steps. The ANOVA thus compares P(BBB) with P(BBT) and correspondingly P(TBB) with P(TBT) to test for precognition effects, while controlling for the immediate perceptual history. The ANOVA further compares P(BBB) with P(TBB) and P(BBT) with P(TBT) to test for history effects, while controlling for possible precognition effects.

Given that the Necker lattice is physically completely ambiguous, we can postulate equal probabilities of p = 0.5 to perceive it in the B- and T-perspective if no history effects (neither short-term nor long-term) and no precognition effects are present (null hypothesis). Systematic deviations from the 0.5 level would be evidence in favor of an a priori perceptual bias for the Necker cube (i.e. a long-term perceptual history effect). The ANOVAs thus further tested for this with the factor BIAS.

## Results–Experiment 1

**Fig 6:**
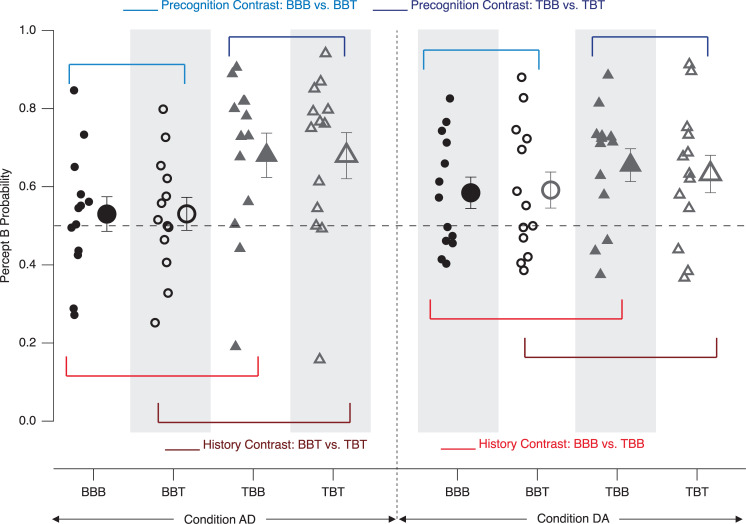
Perceptual probability results–Experiment 1. Depicted are individual probability values as calculated with the formulas (1–4), introduced above. Any systematic deviation from the dashed grey 0.5 line indicates a perceptual bias in the respective direction, i.e. a long-term perceptual history effect. Small icons represent data from individual participants, large icons the respective grand means (± SEM). Blue and red lines indicate the precognition and short-term history effect contrasts, respectively.

**Fig 7:**
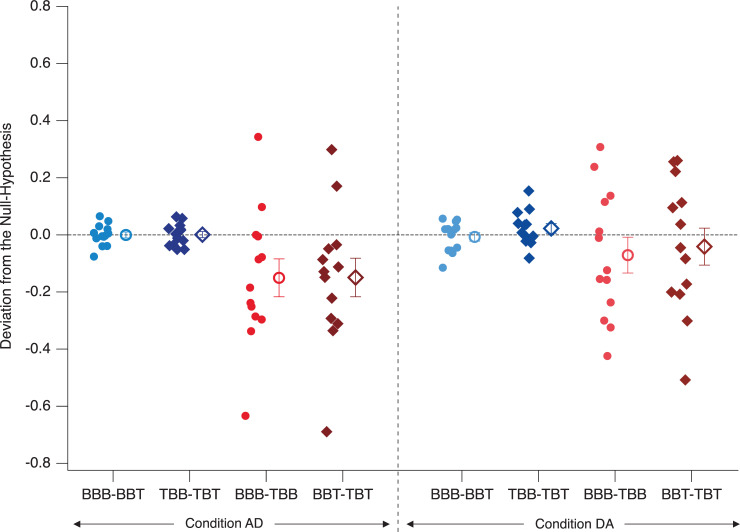
Perceptual history and precognition contrasts. Depicted are individual differential probability value contrasts as indicated on the abscissa. Any systematic deviation from zero indicates either immediate perceptual history effects (red icons) or precognition effects (blue icons). Small icons represent data from individual participants, large icons the respective grand means (± SEM).

**Fig 8:**
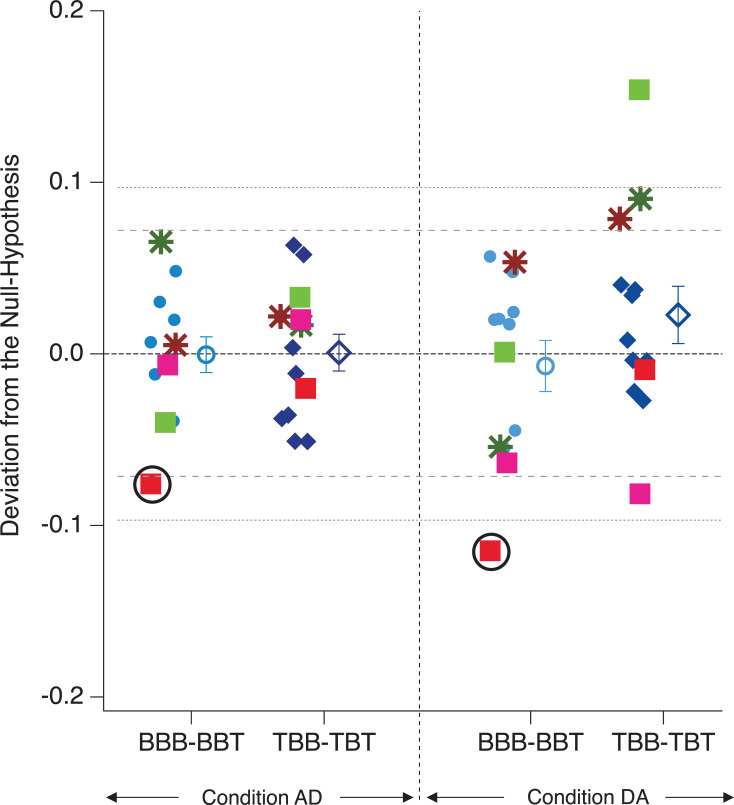
Enlarged precognition contrasts. Depicted are individual differential probability value contrasts (the same blue icons from Fig 7 on an enlarged y-axis). The dashed and dotted grey horizontal lines indicate 1.5 and 2 standard deviations (SDs). All colors other than blue indicate participants with differential probability values equal or larger than 1.5 SD in at least one data set (column). Of notice is the participant indicated by the red square (surrounded by a black circle), who showed such a deviation two times and in the same direction. Filled icons represent data from individual participants, open icons the respective grand means (± SEM).

A graphical demonstration of the different perceptual probabilities on both the level of participants and grand means is presented in Fig 6. The contrasts mentioned above for both the perceptual history and precognition analyses (differences between the respective probability values) are graphically presented in Figs 7 and 8. In these difference graphs, any systematic deviation of the differences of probability values from zero would indicate effects in the respective directions.

Participants perceived the ambiguous Necker lattice overall more often in the B-perspective than in the T-perspective, independent of the identities of the disambiguated preceding and the subsequent lattice variants and of the experimental conditions (p = 2.9*10^−09^, F(1,96) = 42.84, η^2^ = 0.22 for the factor BIAS). Fig 6 indicates this by the fact that the majority of icons are above the (grey dashed) horizontal 0.5-line. Further, in the condition AD the probability to perceive the Necker lattice in the B-perspective was significantly larger, when the preceding stimulus was the disambiguated lattice variant with the T-perspective, than when it was in the B-perspective. This can also be observed as a tendency in the DA condition, but the effect is much weaker (p = 0.003, F(1,96) = 9.27, η^2^ = 0.02 for the interaction between the factors HISTORY and BIAS). This can be seen in Fig 6, where more of the icons in the TBB and TBT sequences are above the 0.5-line than in the BBB and BBT sequences and where this pattern is more prominent in the AD condition (left) than in the DA condition (right).

We found no statistically significant precognition effect, no further difference between the experimental conditions and no further interaction.

## Discussion–Experiment 1

In Experiment 1, we presented ambiguous Necker lattices and disambiguated lattice variants in alternation and investigated whether the perceptual interpretations of the ambiguous Necker lattice can be influenced by the identity of the preceding (history effects) and/or of the subsequent (precognition effects) disambiguated lattice variants.

### History effects

We found strong evidence for long-term perceptual history effects. Participants perceived the ambiguous Necker lattices in general more often in the B-perspective than in the T-perspective, independent of the experimental condition and also independent of the identities of the preceding and subsequent disambiguated lattice variants. This is a confirmation of reports about an a priori bias in favor of the B-perspective, when viewing an ambiguous Necker cube stimulus [9–11,28]. The current (post-hoc) explanation of this effect is that we look much more often down on objects in our environment than up on them. This recurring perceptual experience over lifetime may thus serve as a long-term perceptual memory bias in favor of an a priori from-above perspective (i.e. the B-perspective).

The probability of B-perspective percepts increased even more, when the preceding stimulus was a disambiguated lattice in the T-perspective. However, this short-term perceptual history effect was only present in the Condition AD, but not in the Condition DA (Fig 6: more grey triangular icons are above the 0.5-line on the left than on the right, see also difference values in Fig 7). In the following, we discuss, how these apparently inconsistent results between conditions may be explained in the context of priming and adaptation.

Several studies with ambiguous figures reported both positive (priming) and negative (adaptation) effects of the immediate perceptual history on the current percept [8,10,31,65–72]. A recent study even indicated erroneous percepts of a currently observed stimulus, which could be traced back to the immediate perceptual history [73].

The basic idea of priming in the context of ambiguous figures is, that the disambiguated stimulus pre-activates the related neuronal representation. The pre-activated representation then dominates perception of the subsequent ambiguous stimulus.

The basic idea underlying adaptation is that perceiving the disambiguated stimulus variant for a longer time period reduces the responsiveness and/or stability of the underlying neural representation. At the time point when the ambiguous stimulus variant replaces the disambiguated stimulus, the not-yet adapted neural representation of the alternative stimulus interpretation dominates the adapted one and the observer perceives the ambiguous stimulus in this interpretation. After a previously dominant neural representation has become non-dominant, it starts to recover from adaptation with a stimulus-specific and most probably also participant-specific time constant.

A number of studies are particularly interesting for the present findings, starting with work by Orbach et al. [31], where the authors observed that recovery from adaptation starts immediately after the stimulus is turned off. However, if the next stimulus occurs before recovery has finished, the novel adaptation adds to the remaining level at stimulus onset–and so on (like a saw-tooth, however with an overall increase of adaptation level, until a maximum is reached). In a follow-up study [71], the same group found that, presenting a disambiguated version of the Necker cube, changes the perceptual probabilities of the two interpretations of a subsequently presented ambiguous Necker cube. In another relevant study, Long et al. [8] worked with two separate ambiguous figures, namely with the Necker cube and with overlapping squares. In separate conditions they presented disambiguated variants of these stimuli for varying durations, ranging from 0 to 160 seconds. The disambiguated stimuli were followed by the ambiguous variants and participants indicated their percept of the latter. For short presentation durations of the disambiguated stimulus variants up to about 20 seconds, Long et al. found priming effects, i.e. participants perceived the ambiguous stimulus in the same way as the preceding disambiguated variant. For longer presentation times of the disambiguated stimulus variants, starting at about 80 seconds, they reported adaptation effects, i.e. participants perceived the ambiguous stimulus in opposite orientation as the preceding disambiguated variant. The authors termed this opposite pattern of perception of an ambiguous stimulus as a function of the duration of a previously observed disambiguated stimulus variant as “reverse-bias effect” [74]. In yet another study, Toppino et al. found that the adaptation effect becomes weaker as a function of the duration of a gap between the disambiguated and the ambiguous stimulus [75]. The authors recently replicated their findings and provided an elaborated overview of the topic in the introduction of their publication [76].

The literature above indicates that priming and adaptation are two independent processes that work on different time scales. Further, both neural representations (the B-perspective and the T-perspective) can be adapted independently. Moreover, the impacts of both priming and adaptation start to decay immediately after the conditioning stimulus (in our case the disambiguated lattice) is turned off (and in our case replaced by a grey screen in the ISI and IOSI). In addition, if the next conditioning stimulus occurs before the adaptation state of the perceptual system hasn’t fully recovered, the new adaptation adds upon the remaining adaptation state.

Given this background, one explanation for the heterogeneous results for the immediate history effects in the Conditions AD and DA may be the following: We assume that neither the ISI nor the IOSI are enough time for full recovery of adaptation states. As a consequence, due to the repetitive presentation of the disambiguated stimuli, adaptation of both perceptual representations may cumulatively reach a relatively high level of adaptation. In both conditions priming may also take place, however priming works on a much shorter time scale than adaptation. In Condition AD, the grey screen gap between the preceding disambiguated lattice (i.e. the IOSI, see Fig 5) and the ambiguous lattice is 600 ms longer than in the DA Condition (i.e. the ISI, see Fig 5). The compensatory power of the priming effect (against adaptation) may thus be weaker–due to the stronger decay of priming effects–resulting in a stronger adaptation effect in the AD condition than in the DA condition.

### Precognition

We found no significant precognition effect on the group level. However, some interesting individual patterns of precognition-like effects are indicated in Fig 8: Two participants showed a difference between the compared probability values larger than 0.1 (indicated by green and red squares in Fig 8). Particularly, one participant (the red square, surrounded by a black circle) showed a consistent deviation of the difference value from zero larger than 1.5 standard deviations and with identical signs in both experimental conditions (Fig 8: the BBB minus BBT contrasts in AD and DA; the grey dashed and dotted lines indicate 1.5 and 2 standard deviations, respectively).

The finding of relatively strong group-level history effects for the perception of the ambiguous Necker lattice but no group-level precognition effects, raises questions about the role of the perceptual history for potential precognition effects and whether precognition effects can be identified in an experimental setting without an influential immediate perceptual history as in this Experiment 1. We focused on this question in our Experiment 2.

## Methods–Experiment 2

### Participants

Twenty-one participants (14 women, 7 men) took part in Experiment 2. The median age was 24, ranging from 19 to 31. All participants had normal or corrected-to-normal visual acuity [57] and gave their written consent to participate in this study. The study was performed in accordance with the ethical standards laid down in the Declaration of Helsinki [58] and was approved by the local ethics board.

### Stimuli

We used the same stimuli as in Experiment 1.

### Paradigm

**Fig 9:**
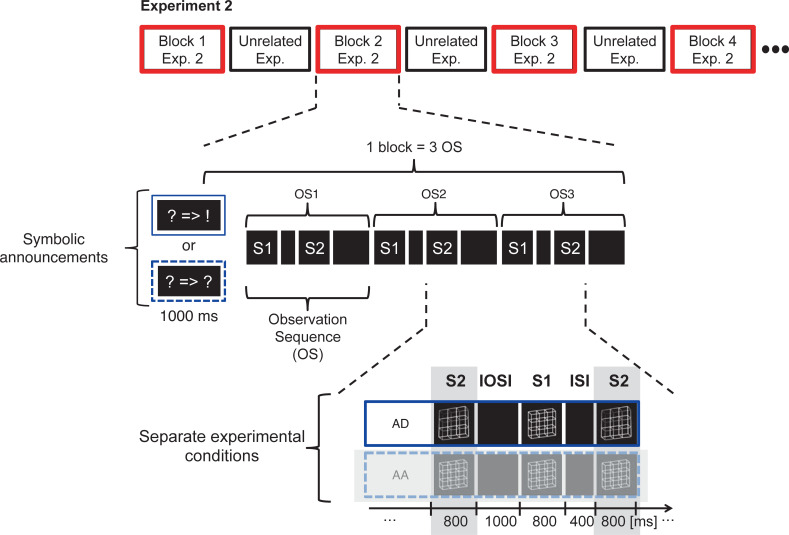
Paradigm of Experiment 2 – schematic overview. Blocks of Experiment 2 (top red framed) were interlaced with blocks of an unrelated experiment (top black frames). Blocks in Experiment 2 consisted of 3 observation sequences (OS1, OS2, OS3). OS1 was preceded by a symbolic announcement of the upcoming experimental condition (in blue rectangles). Experiment 2 consisted of two experimental conditions. Each stimulus pair in Condition AD consisted of an ambiguous lattice (S1) followed by a disambiguated lattice (S2). The stimulus pairs in Condition AA contained only ambiguous lattices. Like in Experiment 1, participants indicated the perceived 3D orientation of lattice S1 (Task 1). After presentation of S2 they compared the perceived 3D orientation of S2 with the memorized percept from S1 and indicated either reversed percepts or perceptual stability by key press (see Fig 2 for the graphical representation of the tasks). Notice, that for the focus of the current analysis we only analyzed Condition AD. A variant of this figure has already been presented in a recent publication from our lab [see Fig 8 in 61].

The paradigm of Experiment 2 was very similar to that of Experiment 1 with the following exceptions:

1. Experiment 2 was executed with a new sample of participants.
2. Experiment 2 contained only Conditions AA and AD. We analyzed the Condition AD.
3. Each experimental block contained only three observation sequences. This reduced the length of an experimental block from about 9 minutes (Experiment 1) to about 9 seconds (Experiment 2). Concurrently, we drastically increased the number of experimental blocks per condition by factor 40 from 3 blocks (Exp. 1) to 120 blocks (Exp. 2). The reason for this change will be explained in the following.
4. At the beginning of each experimental block, we announced the experimental condition to which this block belonged with abstract symbols (see Fig 9).
5. Experiment 2 was interlaced with a second unrelated experiment. This additional experiment also contained experimental blocks of about 9 seconds each, where we presented happy or sad smiley faces. The blocks from Experiment 2 and from the additional experiment alternated with each other (for a schematic overview see Fig 9, top). The basic idea of this interlacing was to eliminate within-experiment visual short-term memories from one block of one experiment to the next of the same experiment. As a result, each first OS in a block (labeled as OS1 in Fig 9) should be free of an immediate experiment-specific perceptual history. OS1 should thus serve to study precognition effects without any influence from the immediate perceptual history. The two subsequent pairs (OS2 and OS3 in Fig 9) may show effects of accumulating experiment-specific entries into short-term memory.

In Experiment 2, the nomenclature of the various sequences (BBB, BBT, etc.) within OS2 and OS3 are identical to those of Experiment 1. For OS1, however, no directly preceding disambiguated stimulus exists. Therefore, the sequences in OS1 are sorted and labeled in the following way:

BB:

- The currently observed ambiguous lattice S1 from the current OS is perceived in the **B**-perspective.
- The disambiguated lattice S2 from the current OS will have a **B**-perspective.

BT:

- The currently observed ambiguous lattice S1 from the current OS is perceived in the **B**-perspective.
- The disambiguated lattice S2 from the current OS will have a **T**-perspective.

According to this rule the other two groups were TB and TT.

### Data analysis

We calculated a separate precognition analysis for OS1, comparing the probability of perceiving the ambiguous Necker lattice in the B-perspective as function of a subsequent disambiguated lattice also in the B-perspective versus a subsequent disambiguated lattice in the T-perspective. To do so, we performed classical repeated-measures t-tests and for confirmation purposes additional Wilcoxon tests.

For the OS2 and OS3 we calculated a repeated-measures ANOVA with the factors (immediate) HISTORY, (immediate) FUTURE and OBSERVATION SEQUENCE, each of these factors containing two factor steps. As in the analysis from Experiment 1, the ANOVA further tested the individual probabilities against p = 0.5 (factor BIAS).

## Results–Experiment 2

**Fig 10:**
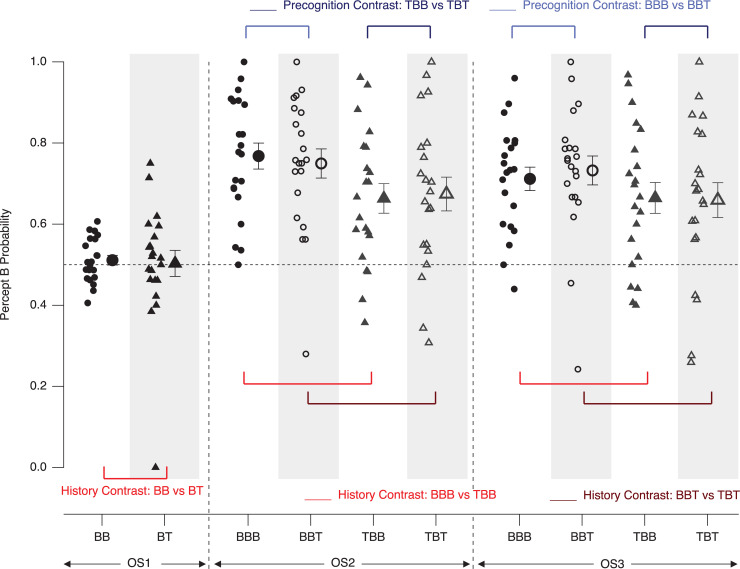
Perceptual probability results– Experiment 2. Depicted are individual probability values, as calculated with the formulas (1–4), introduced above. Any systematic deviation from the dashed grey 0.5 line indicates a perceptual bias in the respective direction (long-term perceptual history effect). Small icons represent data from individual participants, large icons the respective grand means (± SEM). Blue and red lines indicate precognition and history contrasts, respectively.

**Fig 11:**
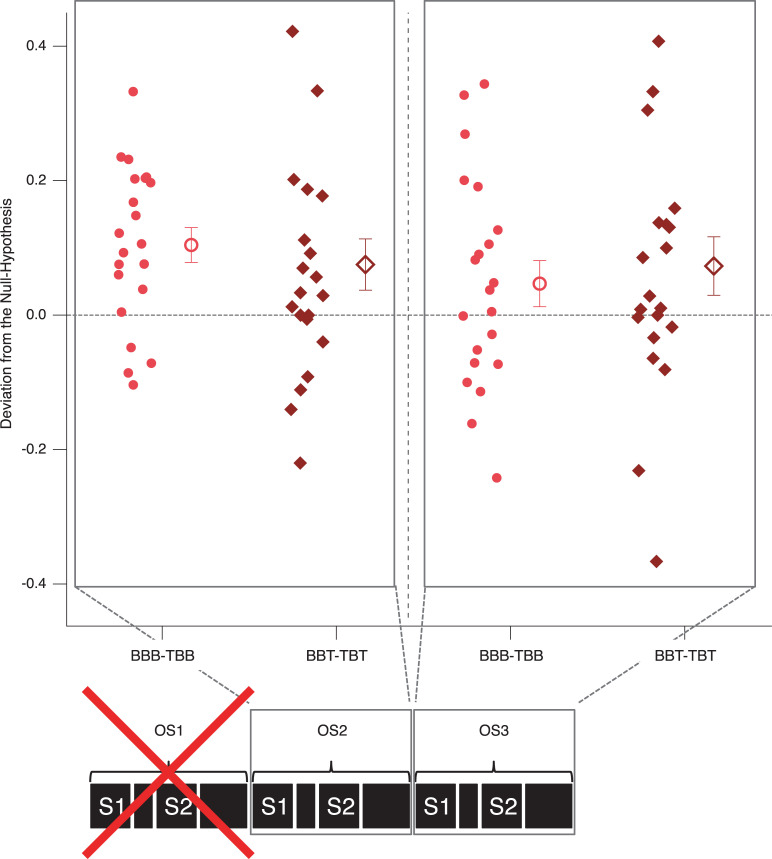
Perceptual history contrasts– Experiment 2. Depicted are individual differential probability value contrasts, as indicated on the abscissa. Any systematic deviation from zero indicates an effect of the immediate perceptual history on the perception of the ambiguous Necker lattice. Filled icons represent data from individual participants, open icons the respective grand means (± SEM). Remarkably, the history effects in this experiment had an opposite effect (priming) compared to Experiment 1. Notice that OS1 had no stimulus-specific perceptual history and was thus not analyzed in this way. OS = observation sequence.

A graphical demonstration of the different perceptual probabilities on both the level of participants and grand means is presented in Fig 10. Fig 11 provides difference values subtracting the specific probability values from each history and precognition contrasts. In these graphs, any systematic deviation of the differences of probability values from zero would indicate effects in the respective directions. Table 3 provides the averages of the numbers of sequences per participant that entered the analyses.

**Table 3:** Average numbers of sequences.

	**OS1**	
**BB**	44.14 (±1.64)	
**BT**	42.76 (±2.25)	
**TB**	14.19 (±1.69)	
**TT**	13.76 (±1.72)	
	**OS2**	**OS3**
**BBB**	22.43 (±1.1)	21.43 (±0.89)
**BBT**	23.05 (±1.4)	20.95 (±1)
**TBB**	19.91 (±1.3)	19.33 (±1.44)
**TBT**	19.38 (±1.45)	19.57 (±1.38)
**BTB**	7.1 (±1.05)	8.76 (±0.88)
**BTT**	7.43(±0.97)	8.05 (±1.21)
**TTB**	10.19 (±1.17)	9.71 (±1.26)
**TTT**	9.29 (±1.25)	10.19 (±1.28)

Average number of sequences across participants (± SEM).

Overall, both the ANOVA results and Fig 10 indicate a strong perceptual long-term history effect: The majority of icons in OS2 and OS3 are above the dashed horizontal 0.5 probability line. This means that the participants tend to perceive the Necker lattice more often in the B-perspective than in the T-perspective, independent of the identity of the preceding and the subsequent disambiguated lattice stimulus, as is also indicated by the ANOVA results (p = 2*10^−16^, F(1,160) = 243.92, η^2^ = 0.6). This effect was even stronger if the preceding disambiguated lattice had been presented in the B-perspective (p = 0.019, F(1,20) = 6.5, η^2^ = 0.24 for the factor HISTORY), compared to disambiguated predecessor lattices in the T-perspective. The ANOVA indicated no effects for the factor OBSERVATION SEQUENCE and no interaction between HISTORY and OBSERVATION SEQUENCE.

The ANOVA further indicated no effects for the factors HISTORY and OBSERVATION SEQUENCE and no related interactions.

Interestingly, the separate analysis of OS1 indicated neither a history effect nor a precognition effect.

**Fig 12:**
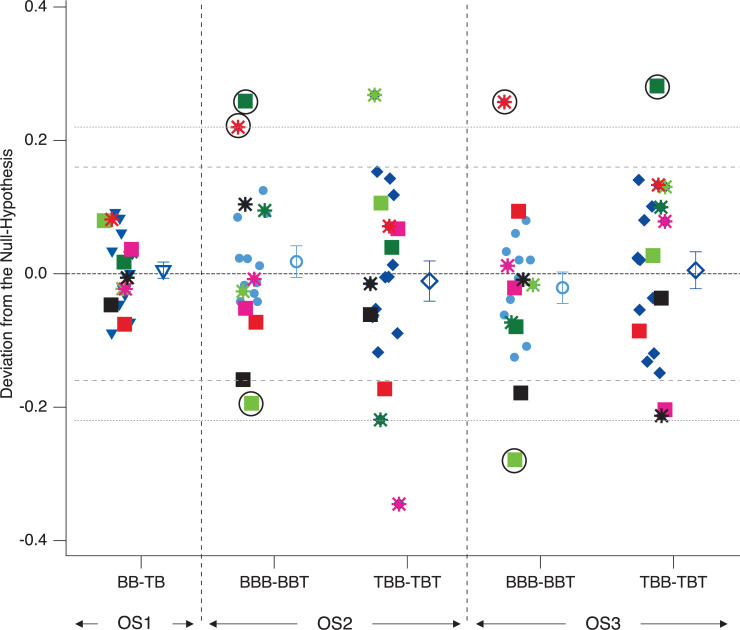
Precognition contrasts– Experiment 2. Depicted are individual differential probability value contrasts, as indicated on the abscissa. Any systematic deviation from zero would indicate a precognition effect. Filled icons represent data from individual participants, open icons the respective grand means (± SEM). We found no significant precognition effects. However three participants (indicated with a red star and light and dark green squares and surrounded by black circles) show systematically extreme values–exceeding 1.5 SDs–across different OS and data sets. Moreover, post-hoc analyses indicated smaller intra-group variability for OS1 (BB-BT) compared to all other data sets (see also Table 2).

Like in Experiment 1, despite no significant precognition effect on the group level (factor FUTURE), we found some interesting precognition-like patterns on the level of individual participants (Fig 12).

Moreover, closer inspection of Fig 12 revealed some interesting variability patterns with smaller within-group variability in OS1 compared to OS2 and OS3. We thus performed exploratory post-hoc Barlett-tests between the data set from OS1 and the data sets from OS2 and OS3. We also performed a Barlett-test comparing the data sets from OS2 and OS3 with each other. The results are presented in Table 4 and indicate significantly smaller variance in the data set from OS1 compared to all other data sets. Further, the variability of the data sets from OS2 and OS3 do not differ between each other.

**Table 4:** Barlett tests.

Barlett Test	Calculated Value	Critical Value	p <
**OS1(BB-BT) vs. OS2(BBB-BBT)**	8.18	6.63	0.01
**OS1(BB-BT) vs. OS2(TBB-TBT)**	14.35	10.83	0.001
**OS1(BB-BT) vs. OS3(BBB-BBT)**	8.01	6.63	0.01
**OS1(BB-BT) vs. OS3(TBB-TBT)**	11.96	10.83	0.001
**OS2(BBB-BBT) vs. OS2(TBB-TBT) vs. OS3(BBB-BBT) vs. OS3(TBB-TBT)**	2.15355	2.71878	Ns

## General discussion

In Experiment 1, we found statistical evidence for large long-term perceptual history effects (the a priori bias) and smaller short-term perceptual history effects (influence of the immediate perceptual history). No statistically significant precognition effect was indicated, but some interesting precognition-like patterns on the level of individual participants could be observed.

In Experiment 2, we modified the paradigm from Experiment 1 to test for potential precognition effects in a situation without a stimulus-specific perceptual history (no immediately preceding stimulus-specific OS) and with a slowly accumulating perceptual history (one preceding OS and two preceding OS). We found again strong long-term perceptual history effects and weaker short-term perceptual history effects for OS2 and OS3, i.e. in the case of one and two preceding OS. Interestingly, the observed short-term perceptual history effects from Experiment 2 had an opposite sign (priming) compared to the short-term perceptual history effects from Experiment 1 (adaptation) (compare Figs 7 and 11).

Again, we found no statistically significant precognition effects in OS2 and OS3 of Experiment 2, but some interesting patterns on the level of individual participants. In this Experiment 2, we particularly focused on OS1, the only OS without a perceptual short-term history of potentially influential disambiguated lattice variants. Also this OS1 revealed no significant precognition effect, regarding perceptual probability values. However, in an exploratory additional analysis we found smaller within-group variability for the data set of difference values from OS1 compared to the data sets from OS2 and OS3.

### Potential explanations of the pattern of perceptual history effects

At first glance, the opposite pattern of short-term perceptual history effect findings from Experiments 1 and 2 seem to be contradictory and puzzling and suggest type-1-errors rather than substantial effects. However, these results can be easily integrated into the existing literature. Already in the Discussion section of Experiment 1 we introduced literature findings about priming and adaptation effects during observation of ambiguous figures. Xx no return hereThe priming effects found in OS2 from our Experiment 2 (perceiving the ambiguous Necker lattice more often in the B-perspective if the preceding disambiguated variant had been presented in this B-perspective), may result from a kind of “beneficial” pre-activation of the perceptual representation, evoked by the preceding perception of the respective disambiguated lattice variant from OS1, which was presented for 800 ms. In line with Long et al.’s findings, 800 ms is short enough to induce such priming effects.

The presence of the adaptation-like history effects in Experiment 1 (perceiving the ambiguous Necker lattice more often in the B-perspective if the preceding disambiguated variant had been presented in the T-perspective) can be explained in a similar vein. The blocks in Experiment 1 lasted much longer and contained a much larger number of successive OS. Given that the whole Experiment 1 lasted for about 1.5 hours, the data sets contained more trials with long (> 80 seconds) than with short perceptual histories (< 20 seconds). Keep in mind that the perceptual adaptation theories postulate that each neural representation (here the T- and the B-perspectives) can be adapted independently (e.g. [31,65]). We thus assume that in Experiment 1 both neural representations became adapted to a certain and probably comparably large degree over a certain number of presentations of the respective stimuli. In the highly adapted states, the immediate disambiguated lattice antecessor may then tip a given adaptation balance in the respective direction.

This postulated explanation can unfortunately not be tested with the present data sets, because we do not have enough trials to also take into account the perceptual histories at earlier time points t_(-2)_, t_(-3)_ etc. Such an extended analysis would increase the number of data sets that have to be separated from each other. This, in turn, would reduce the number of trials per data set dramatically, making reliable statistics impossible.

Further, it was found that not only the preceding stimulus identity, but also knowledge about the occurrence probabilities of previously presented stimuli influence the current perceptual interpretation [77]. Current models of perception, like Bayesian probability [29], predictive coding [2] or circular inference models [78] postulate that past perceptual experiences are used to make predictions of upcoming sensory information and highlight the influence of this integration on current perceptual processes [for electrophysiological correlates see 64]. These theories suggest a highly complex and hierarchical network of predictive influences from the perceptual past on the perceptual presence and a full experimental control of all such aspects is a real challenge.

### Discussion of the heterogeneous precognition-like patterns

One critical point in precognition research and also more general in research about extrasensory perception or psychokinesis experiences ("PSI": the causes underlying extrasensory perception and psychokinesis experiences cannot be explained by known physical or biological mechanisms [79]) is the recurring pattern that positive study findings cannot be reliably replicated [42,47–50,80]. One attempt to explain these replication problems refers to the possibility that only some particularly gifted persons show large PSI effects, while the majority of participants do not [e.g. 81]. Theoretically assuming that this is the case and additionally assuming that such gifted persons are a priori rare, any study that randomly collects participants will be convicted to produce either non-significant or only weakly significant results on the group level. Taking this option into account, we identified potentially gifted “outlier participants” with maximal deviations of the calculated difference values from zero (above 1.5 and 2 standard deviations, “SD”) in each of the analyzed data sets and checked whether they systematically show extreme values also in the other analyzed data set.

In Experiment 1, we identified one outlier participant (red square in Fig 8), who in both Conditions AD and DA in the “BBB minus BBT” showed differences of probability values with the same sign and both above 1.5 SD (0.08 in Condition AD and about 0.12 in Condition DA).

A similar pattern was found in Experiment 2 for the observation sequences OS2 and OS3. Two participants in this experiment showed consistent deviations of their differential probability values of about 0.22 or larger (red star and dark green square in Fig 12) across two separate contrasts. Again, both were above the 1.5 SD line. While for one participant (red star in Fig 12) this deviation was observed in the same contrast (BBB-BBT) over observation sequences OS2 and OS3, the contrast type changed from BBB-BBT (OS2) to TBB-TBT (OS3) for the other participant (dark-green square in Fig 12).

Even if we assume the correctness of the null hypothesis of no precognition effects, we should expect some variability of the data from individual participants among the expected zero mean (Figs 8 and 12). Yet we should not expect the repetitive pattern of individual participants as described above. However, the main focus of our Experiment 2 was on OS1, where we tried to reduce the potential influence from the perceptual history as a potentially confounding factor for the precognition analysis as much as possible. It turned out that the three remarkable outlier participants across OS2 and OS3 were unremarkable in the OS1 where the influences of perceptual history were minimized. This, in turn, indicates that the perceptual history is the most probable causal factor for the pattern these special participants show in OS2 and OS3, even though it will be difficult to explain how this influence may unfold in these cases [but see 82 for a speculative alternative explanation].

Fig 12 provides another interesting and related pattern: The variability of probability difference values was much smaller in OS1 (no immediate perceptual lattice history) compared to OS2 and OS3 (accumulating perceptual lattice history), where the variability was similar but clearly larger than in OS1. A post-hoc exploratory analysis indicated statistical significance of these observations (see Table 4). This indicates that the tendency for larger deviations from zero concerning precognition effects seems to be somehow dependent on the existence or non-existence of a stimulus-specific immediate perceptual history.

We regard the observations on the individual level interesting and worth mentioning, although they are not statistically supported and thus do not allow for strong conclusions. We regard the variability effects in Experiment 2 as also highly interesting. The effects are visible in the precognition contrasts, but the given paradigm clearly indicates that the perceptual history plays a role, as already discussed above. Again, the present data do not allow for strong conclusions, but they invite to have a closer look on the variability parameter in future studies.

The literature contains vivid and highly controversial discussions about the question ofwhether it is at all theoretically possible that effects like precognition exist, or whether it is worth or even ethically justifiable to execute related studies [e.g. 83,84]. The aim of the present article is not to contribute to such–in our view–dispensable discussions. Rather, we aim to emphasize the importance of the perceptual history for how we perceive the world at the present moment and how we will perceive it in a future moment. Future precognition studies should thus also take into account that past percepts leave their “footprints” in our perceptual system and some footprints can be deeper than others. The latter can nicely be demonstrated with the a priori perceptual bias of the Necker cube in favor of the B-perspective, what we labeled as the long-term perceptual history effect and what we already described in the introduction. The proposed preference of our perceptual system for the B-perspective most probably reflects the majority of our everyday experiences and can thus be regarded as a “very deep footprint from the past”.

In the present paradigm, it is of course not difficult to comprehend the direct influence from past percepts of a disambiguated lattice figure on the perception of a highly similar but ambiguous lattice variant. In other precognition paradigms, such as some of those used in the experiments of the seminal Bem paper [85], the potential role of the perceptual history is not as directly comprehensible as in the present study– which does not necessarily rule it out. Particularly, given that it is even unclear so far, which factors of past perceptual experiences contribute to the perception of a present sensory information, it will be extremely hard, if not impossible, to exclude all possible influences from the perceptual past on a given precognition effect. In a perfect experiment, one would have to know the history of the participant’s exposure to similar stimuli, the predictive models formed within the participant’s brain and how the sensory information presented within the experiment is represented in the participant. Only in the case of full access to all underlying processes within the participant’s brain, one would be able to doubtlessly disentangle potential influences from the past and potential influences from the future. An additional complication may result from the potential individual differences concerning the amount of impact of past perceptual experiences.

In conclusion, the present study is an example for a central feature of our perceptual system: We prefer to perceive what we already know, and what we currently perceive can strongly influence what we see next. Future studies about perception, whether they regard sensory or extrasensory perception, need to take this into account.
